# What determines the patients’ demand for hospital care services?

**DOI:** 10.1186/1472-6963-14-S2-P137

**Published:** 2014-07-07

**Authors:** Catharina J van Oostveen, Hester Vermeulen, Piet J Bakker, Dirk J Gouma, Dirk T Ubbink

**Affiliations:** 1 Department of Quality Assurance & Process Innovation, Academic Medical Center, Amsterdam, The Netherlands; 2 Department of Surgery, Academic Medical Center, Amsterdam, The Netherlands; 3 Amsterdam School of Health Professions, University of Amsterdam, Amsterdam, The Netherlands

## Background

Hospitals are constantly being challenged to provide high-quality care despite ageing populations, diminishing resources, and budgetary restraints. While the costs of care depend on the patients’ needs, it is not clear which patient characteristics are associated with the demand for care and inherent costs. Therefore, a practical explanatory model would avail healthcare professionals and managers in determining the demand and costs for clinical care. We performed three studies to generate a valid model.

## Materials and methods

First, a systematic review was conducted by searching the databases MEDLINE, Embase, Business Source Premier and CINAHL. Pre-defined eligibility criteria were used to detect studies that explored patient characteristics and health status parameters associated with the use of hospital care services for hospitalized patients. Second, a time and motion pilot-study was performed among surgical wards in a Dutch university hospital. To include a representative sample of patients admitted to clinical wards, the most common diagnoses were selected from the national medical registry of ICD-10 diagnoses. Surgeons and nurses recorded the time spent on patient care 24/7 by means of PDAs. The patients’ total demand for care was expressed as costs of medical and nursing time spent, surgical interventions, and diagnostic procedures. Linear regression analysis was applied to detect significantly contributing characteristics. Third, the same method was applied in a broader setting, comprising three medical specialties in a Dutch university hospital; surgery, paediatrics, and obstetrics & gynaecology.

## Results

From the 2,168 articles potentially relevant for the systematic review, 17 met our eligibility criteria. These showed a large variety of factors associated with the use of hospital care services. Age, gender, medical and nursing diagnoses, severity of illness, patient acuity, comorbidity, and complications were the most commonly found characteristics. For the pilot-study 174 surgical patients were monitored during their hospital stay. Characteristics significantly influencing the consumed amount of care were: number of medications during hospitalisation, complications, co-morbidity, medical specialty, age, as well as undergoing surgery and length of stay. This model was validated by the third study for which 19 surgical, I7 paediatric and 14 gynaecological or obstetric patients were investigated.

## Conclusions

A set of predictors of the demand for hospital care services was found to be valid for different clinical specialties. These factors can all be identified during hospitalization and be used as a managerial tool to monitor the patients’ demand for hospital care services and to detect trends in time.

